# Elucidating novel immune profiles for predicting infection in high‐risk cohorts: a pilot study in patients with relapsed and refractory chronic lymphocytic leukaemia

**DOI:** 10.1002/cti2.70049

**Published:** 2025-08-03

**Authors:** Lewis J Williams, Connie SN Li‐Wai‐Suen, Alex L Garnham, Stefanie M Bader, Constantine S Tam, Ashley Whitechurch, Monica A Slavin, Marcel Doerflinger, Benjamin W Teh

**Affiliations:** ^1^ The Walter and Eliza Hall Institute for Medical Research Parkville VIC Australia; ^2^ Department of Medical Biology The University of Melbourne Melbourne VIC Australia; ^3^ The Alfred Hospital Melbourne VIC Australia; ^4^ Faculty of Medicine, Nursing and Health Sciences Monash University Clayton VIC Australia; ^5^ Department of Clinical Hematology The Royal Melbourne Hospital and Peter MacCallum Cancer Centre Melbourne VIC Australia; ^6^ Department of Infectious Diseases Peter MacCallum Cancer Centre Melbourne VIC Australia; ^7^ Sir Peter MacCallum Department of Oncology University of Melbourne Melbourne VIC Australia; ^8^ National Centre for Infections in Cancer Peter MacCallum Cancer Centre Melbourne VIC Australia; ^9^ Victorian Infectious Diseases Service Royal Melbourne Hospital Melbourne VIC Australia

**Keywords:** chronic lymphocytic leukaemia, immunity, infection, prediction, profiling, risk

## Abstract

**Objectives:**

Chronic lymphocytic leukaemia (CLL) patients are at increased risk for infection, with the risk even higher for relapsed and refractory patients. Clinical assessment of infection risk is increasingly challenging in the era of immune‐based therapies, such as Bruton's tyrosine kinase inhibitors. A pilot study was conducted to elucidate possible predictive immune markers.

**Methods:**

Patients with relapsed and refractory CLL treated with ibrutinib were evaluated. Peripheral blood mononuclear cells (PBMCs) collected at defined intervals (baseline, 3‐ and 6 months following commencement of ibrutinib) were analysed, with or without phorbol myristate acetate (PMA)/ionomycin stimulation, using Luminex and RNA sequencing. Luminex and gene expression profiles were compared between patients that who did and did not develop infection to identify immune signatures associated with infection over a subsequent 3‐month period.

**Results:**

Twenty‐eight patients were included in this pilot study. Forty‐six per cent of patients developed an infection (13 patients, 17 events) over 9 months of patient monitoring. Most infections were clinically diagnosed (72.7%) with the remainder microbiologically diagnosed bacterial (18.1%) and viral (9.2%) infections. Cell populations did not correlate with subsequent infection. An inflammatory transcriptome profile at 3 months following ibrutinib was associated with a subsequent infection episode. Increased whole protein response to PMA stimulation at 3 and 6 months was associated with subsequent risk for infections. Increased whole protein response to PMA stimulation was associated with subsequent risk of infection 3 months after commencing ibrutinib.

**Conclusion:**

The combination of protein and RNA analysis can provide further insight into the interactions of immunotherapies and immunity but should be validated further in large cohorts.

## Graphical abstract

In the present study, we identified several unique protein and RNA signatures that could be used to predict infection in relapse and refractory patients. We highlight the need for comprehensive immune profiling of high‐risk cancer cohorts.

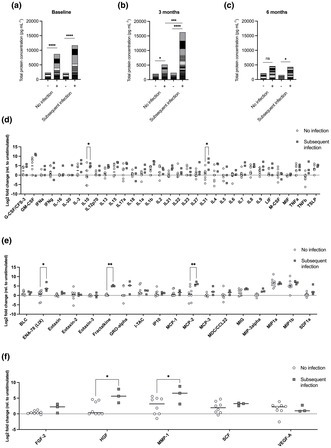

## Introduction

Chronic lymphocytic leukaemia (CLL) represents the most common leukaemia amongst adults in Western Countries.^1^ Whilst this malignancy has been historically treated with chemoimmunotherapy, targeted therapies, such as Bruton's tyrosine kinase (BTK) inhibitors, including ibrutinib, have substantially improved patient outcomes.^2^ These therapies result in depletion of malignant B cells but affect the immune system more variably, with off‐target effects documented on remaining B cells, macrophages, neutrophils and CD4 T cells.^3^

With patients living longer and the nature of these therapeutics, infections remain a leading cause of morbidity and mortality.^2^, ^4^ These infection rates tend to be highest within the first 12 months of commencing therapy, and rates are even higher in the setting of relapsed or refractory disease.^5^, ^6^ Compounding this problem, clinical risk assessment for infections has become increasingly complex because of the less predictable interaction between patient, disease and treatment‐related risk factors. As such, it remains challenging to identify high‐risk CLL patients who require preventative measures, such as antimicrobial prophylaxis.

Comprehensive immune profiling of patients with haematological malignancy could enable personalised prediction of infection risk to enable optimal prevention strategies. A recent study associated blood parameters, such as immunoglobulin with risk of infection, and incorporated this into more complex machine learning algorithms for clinical application. However, this technology comes with inherent disadvantages, such as the task of incorporating machine learning into a healthcare system, a precision of 72% and a recall of 75%, and requires 84 different health variables collected over 7 years of patient history.^7^ We propose an alternative approach, utilising immune stimulation, multiplex ELISA protein analysis and RNA sequencing. Previous studies have identified potential immune variables associated with increased risk of infection in patients with multiple myeloma (MM).^8^, ^9^ For example, a Th2‐dominant cytokine response, detected after *in vitro* mitogen stimulation, was associated with an increased risk of infection in the following 3‐month period. In particular, IL‐3 and IL‐5 responses to phorbol myristate acetate (PMA) antigen stimulation were a key predictor of infection risk.^9^ A subsequent study reiterated the correlation between a dominant Th2 response to pro‐inflammatory stimulus and the increased risk of infection.^8^

These identified signatures are unlikely to be universal across other haematological malignancies and/or treatments. Given the success of immune profiling in MM pilot studies, we aimed to utilise the same approach specifically in patients with relapse or refractory CLL treated with BTK inhibitors, in this instance ibrutinib, to identify immune profiles associated with future infection risk as proof of concept in this patient group.

## Results

### Patient characteristics, episodes of infection and outcomes

Samples were available for 26 patients in this pilot study. Twelve patients had samples prior to treatment and constituted the baseline control group. Another 12 patients had samples at 3 months and 12 patients at 6 months following commencement of ibrutinib. For the control group, median age was 79.5 (63.25–84.75) with 25% receiving anti‐CD20 treatment within the last 12 months. Patients with samples at 3 and 6 months had similar demographic, disease and treatment characteristics as the control group, as summarised in Table 1 (all *P* > 0.05). Within 9 months of patient monitoring, 46% of patients developed an infection (11 infective episodes). Severe infections (grade 3 and above) accounted for 18.1% of infective episodes. Most infections were clinically diagnosed (72.7%), followed by MDI bacterial (18.1%) and MDI viral (9.2%). The majority of infections involved the respiratory tract (54.5%), followed by skin and soft tissue (36.3%). Reassuringly, these study demographics and rates of infection are consistent with the broader CLL patient population.^10^, ^11^, ^12^

**Table 1:** Patient demographics

	Baseline CLL population	Ibrutinib‐treated cohort		*P‐*value
		At 3 months	At 6 months	
Sample size	9	11	11	
Age in years (IQR)	79.50 (63.25–84.75)	79.00 (75.00–82.50)	77.50 (75.25–82.50)	> 0.05
Male	6 (66.66%)	9 (81.81%)	8 (72.72%)	> 0.05
Infectious episodes^a^ (infection within 3 months following sample collection)	3 (25.00%)	3 (25.00%)	5 (41.66%)	> 0.05
Charlson Comorbidity Index (IQR)	4 (2–4)	4 (4–4)	5 (5–5)	> 0.05
Previous lines of therapy (IQR)	1.25 (1.00–2.00)	2.00 (2.00–2.00)	2.75 (2.75–2.75)	> 0.05
17p deletion	3 (25.00%)	5 (41.66%)	4 (30.00%)	> 0.05
Anti‐CD20 within 12 months	3 (25.00%)	3 (25.00%)	4 (30.00%)	

^a^ Infection in the 3 months *following* sample collection not at point of collection.

### Impact of ibrutinib on cell numbers and response to stimulation

Immune cell subset analysis of bulk RNA transcripts of peripheral blood mononuclear cells (PBMCs) using dtangle^13^ identified no differences in relative cell populations observed at 3 or 6 months of ibrutinib compared to baseline (Supplementary figure 1; *P* > 0.05). Furthermore, there were no significant differences amongst subpopulations of B cells, T cells, NK cells, macrophages or dendritic cells (*P* > 0.05).

**Figure 1:**
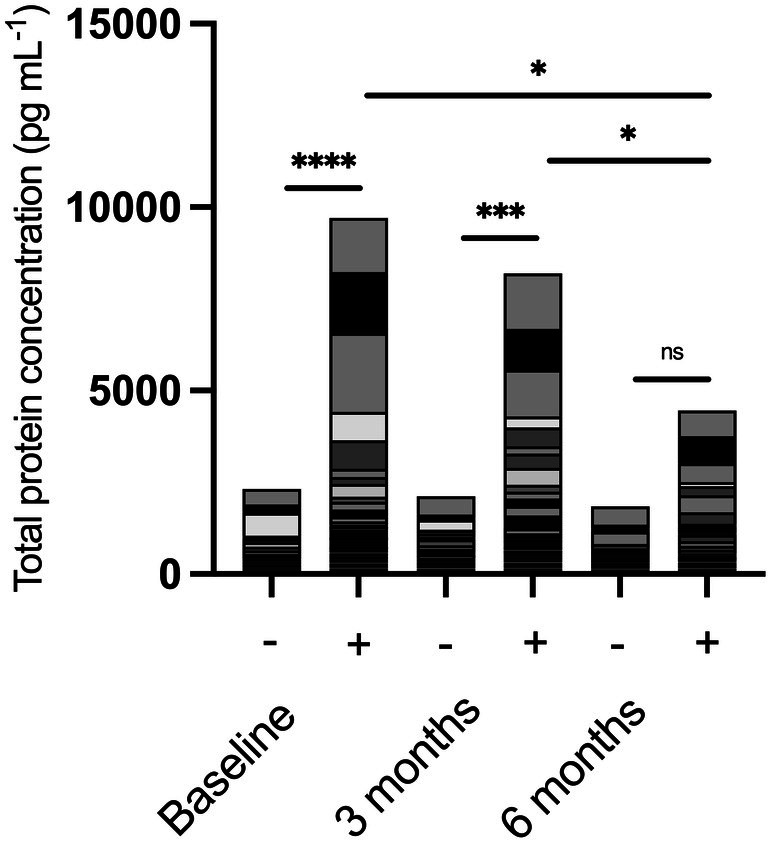
Whole protein profiles in unstimulated (−) and PMA/ionomycin stimulated (+) samples before (baseline) or 3 and 6 months after commencing ibrutinib therapy. Protein expression was determined using a 65‐plex Luminex. Data are presented as accumulative mean, *, *** and **** represent *P* < 0.05, *P* < 0.01, *P* < 0.001 and *P* < 0.0001, respectively. Baseline *n* = 9, 3 months *n* = 11, 6 months *n* = 11.

To understand whether longitudinal ibrutinib treatment impacts the immune system's ability to mount adequate responses, we stimulated the patient‐derived PBMCs *in vitro* with mitogen stimulus of PMA and ionomycin and measured immune‐related protein secretion. Using an ELISA panel of 65 proteins to quantify total accumulated immune response, baseline patient samples responded with significantly increased cumulative protein production by almost 4.2‐fold from 2320 to 9700 pg mL^−1^ (Figure 1; *P* < 0.0001). At 3 months, stimulation significantly increased protein levels by over 3.8‐fold from 2116 to 8197 pg mL^−1^ (*P* = 0.0005). However, this difference in response was lost by 6 months (1848–4459 pg mL^−1^; *P* > 0.05). Additionally, the overall magnitude of the PMA/ionomycin‐induced response was significantly lower at 6 months than at baseline (*P* = 0.0064) and at 3 months (*P* = 0.0421).

**Figure 2:**
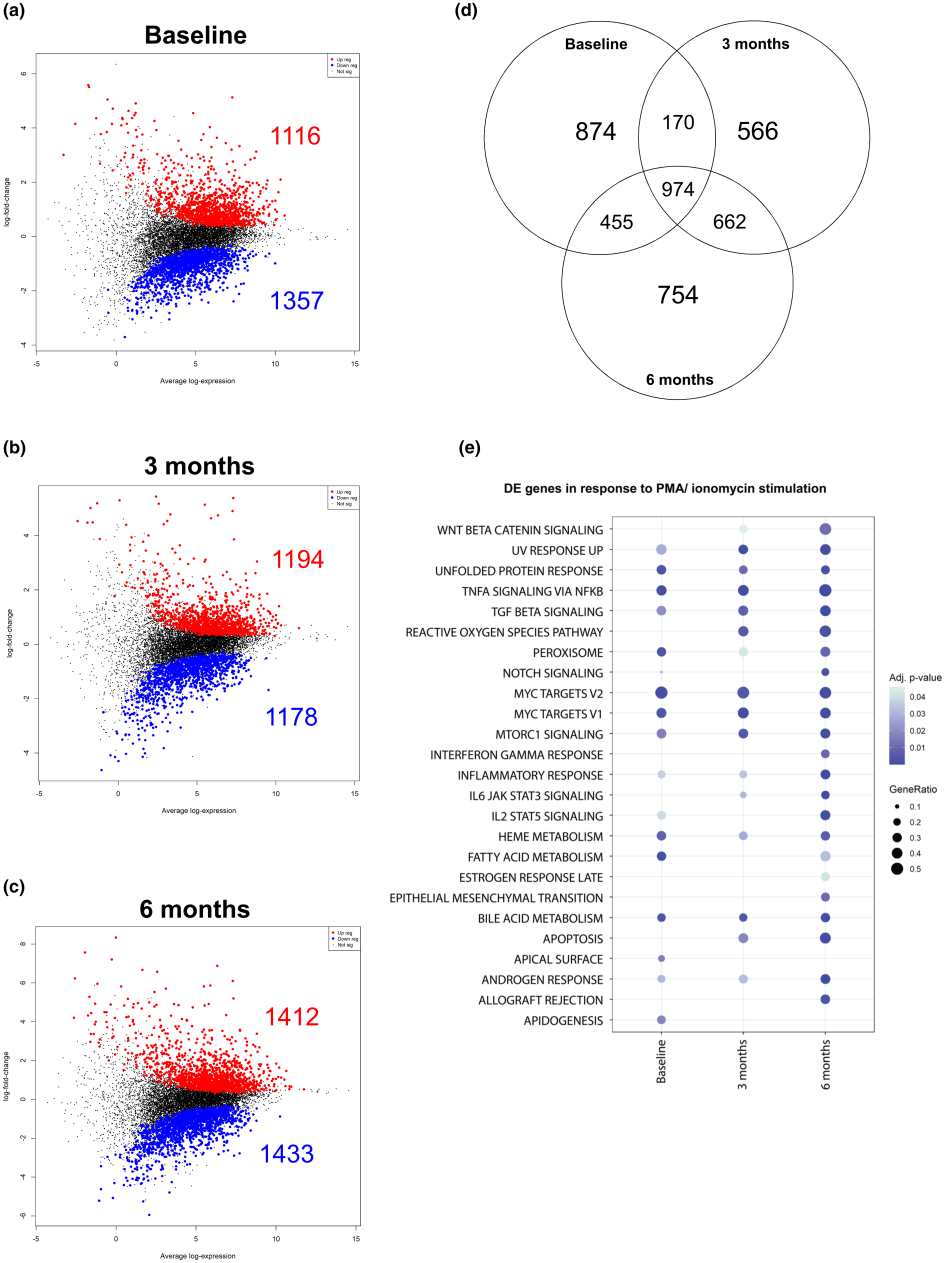
Mean difference plots at baseline **(a)**, at 3 months **(b)** and 6 months **(c)** of PMA/ionomycin stimulated samples compared to respective unstimulated samples. Upregulated genes are represented in red while downregulated genes are in blue. The Venn diagram **(d)** represents differentially expressed genes (DEGs) shared between timepoints. Genes were considered differentially expressed based on an adjusted *P*‐value < 0.05. **(e)** A dot plot demonstrating gene ratios defined by the proportion of DE genes (in response to stimulation) in a hallmark gene set. A hallmark gene set was included if it was significant in at least one time point. The analysis was visually restricted to the top 25 hallmarks (Full list: Supplementary table 3), where significance is denoted by an adjusted *P*‐value < 0.05. Baseline *n* = 9, 3 months *n* = 11, 6 months *n* = 11.

RNA sequencing was conducted on these same PBMC samples to first determine the effects of ibrutinib over time on the transcriptional activity in native state (unstimulated baseline compared to unstimulated 3 and 6 months) of samples, and then on the ability of samples to respond to PMA and ionomycin stimulation after 3 and 6 months of therapy (compared to unstimulated; Figure 2a–c). There was only a marginal impact of ibrutinib on unstimulated samples, with differential expression only occurring at 3 months. This included the downregulation of 369 genes across spermatogenesis, protein secretion, PI3K AKT MTOR signalling and G2M checkpoint hallmarks (data not shown; Supplementary table 2). At baseline, PMA/ionomycin stimulation upregulated multiple inflammatory pathways (Figure 2a), including the upregulation of 1116 genes and downregulation of 1357 genes. At 3 months (Figure 2b), there were 1194 upregulated genes and 1178 downregulated genes, while at 6 months (Figure 2c), 1412 genes were upregulated while 1433 were downregulated. When overlapping the differential gene expression from each timepoint (Figure 2d), there were 974 genes that remained consistent across all three timepoints and 662 differentially expressed genes (DEGs) were shared across 3 and 6 months. Pathway analysis of DEGs over time revealed consistent upregulation of inflammatory hallmarks, such as TNFa signalling, TGF signalling, MYC targets V1 and V2 and MTORC1 signalling. Other pathways such as reactive oxygen species, apoptosis IFNg, WNT beta catenin signalling, late oestrogen response and epithelial mesenchymal transition were only differentially expressed 3 and 6 months after commencing ibrutinib therapy. Apical surface and adipogenesis were differentially expressed at baseline but not after commencing ibrutinib therapy (Figure 2e; full list Supplementary table 3).

### Immune profiling to predict subsequent infection risk

Following this, we investigated the potential of cell populations and response to stimulus as a predictor of subsequent episodes of infection. Patient samples were divided into two groups: those who developed infection within the 3 months following sample collection and those who did not. There were no statistical differences in relative abundance of any cell type that could predict subsequent risk of infection (Supplementary figure 2; *P* > 0.05).

**Figure 3:**
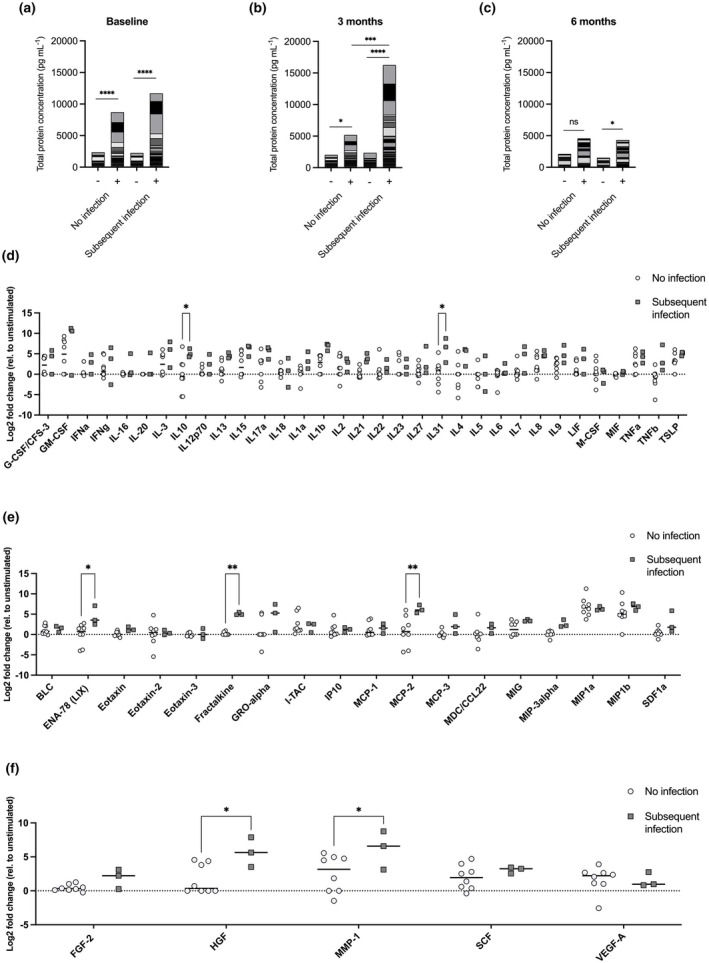
Whole protein profiles in unstimulated (−) and PMA/ionomycin stimulated (+) samples before baseline **(a)** or 3 **(b)** and 6 months **(c)** after commencing Ibrutinib therapy. Protein expression was determined using a 65‐plex Luminex. Data are represented as accumulative mean with *, **, *** and **** representing *P* < 0.05, *P* < 0.01, *P* < 0.001 and *P* < 0.0001, respectively, *n* = 12. Cytokine **(d)**, chemokine **(e)** and growth factors/regulators **(f)** profiles, at 3 months after commencing ibrutinib, in correlation with subsequent infection. Data points represent fold change of PMA/ionomycin stimulation to unstimulated. Protein expression was determined using a 65‐plex Luminex. Data are presented as mean with * and ** representing *P* < 0.05 and *P* < 0.01, respectively. Baseline: infection‐free *n* = 6, infection *n* = 3. 3 months: infection‐free *n* = 8, infection *n* = 3. 6 months: infection‐free *n* = 6, infection *n* = 6.

At baseline (Figure 3a), PMA/ionomycin stimulation elicited significantly increased total protein response compared to unstimulated samples (*P* < 0.0001) but could not differentiate subsequent infection (*P* > 0.05). After 3 months of therapy (Figure 3b), an increase in total protein in response to stimulation was again observed (no infection; *P* = 0.0294 and subsequent infection; *P* < 0.0001). However, this stimulated response increased by over threefold (from 5175 to 16 256 pg mL^−1^) in association with subsequent infection compared to stimulated responses seen in patients who did not develop infection (*P* = 0.0008). At 6 months (Figure 3c), the total protein response induced by PMA/ionomycin did not differentiate infection (*P* > 0.05); however, there was a significant increase in protein response compared to unstimulated controls unique to patients who went on to develop infection (2.8‐fold, 1521 to 4329 pg mL^−1^; *P* = 0.0478).

Given whole protein profiles could be used to predict subsequent infection in ibrutinib patients at 3 and 6 months, we grouped the proteins into four: cytokines, chemokines, soluble receptors and growth factors/regulators to identify more specific correlative signatures. In the previous analysis (Figure 3a–c), the difference between unstimulated and PMA/ionomycin stimulation gave the greatest predictive ability for infection. Therefore, for this analysis, we plotted the fold change difference of PMA/ionomycin stimulation compared to unstimulated samples. There were no differences at 6 months (*P* > 0.05, data not shown). However, at 3 months, increased fold change of cytokines (Figure 3d) IL‐31 (*P* = 0.0135) and IL‐10 (*P* = 0.0365) were correlated with subsequent infection. Furthermore, increased fold change in the chemokines (Figure 3e) ENA‐78 (*P* = 0.0226), fractalkine (*P* = 0.0052) and MCP‐2 (*P* = 0.0011) and growth factors (Figure 3f) HGF (*P* = 0.0197) and MMP‐1 (*P* = 0.0407) were also correlated with subsequent infection. There were no significant correlations for soluble receptors (*P* > 0.05, data not shown).

Given the above data suggested that protein responses could be used as a predictive biomarker, we investigated the potential for unbiased RNA sequencing profiling to predict the risk of infection.^8^ Baseline, 3‐month and 6‐month samples with or without PMA/ionomycin stimulation were compared between patients who developed subsequent infection and those who did not. In unstimulated samples at baseline, no genes were upregulated, while five genes were downregulated in association with subsequent infection. However, there were no DEGs after PMA/ionomycin stimulation. Similarly, at 6 months, there was only one gene upregulated in unstimulated samples, but no differential expression in stimulated samples. Notably, there were no significantly dysregulated pathways that could be correlated to subsequent infection in patients at both these timepoints (data not shown).

**Figure 4:**
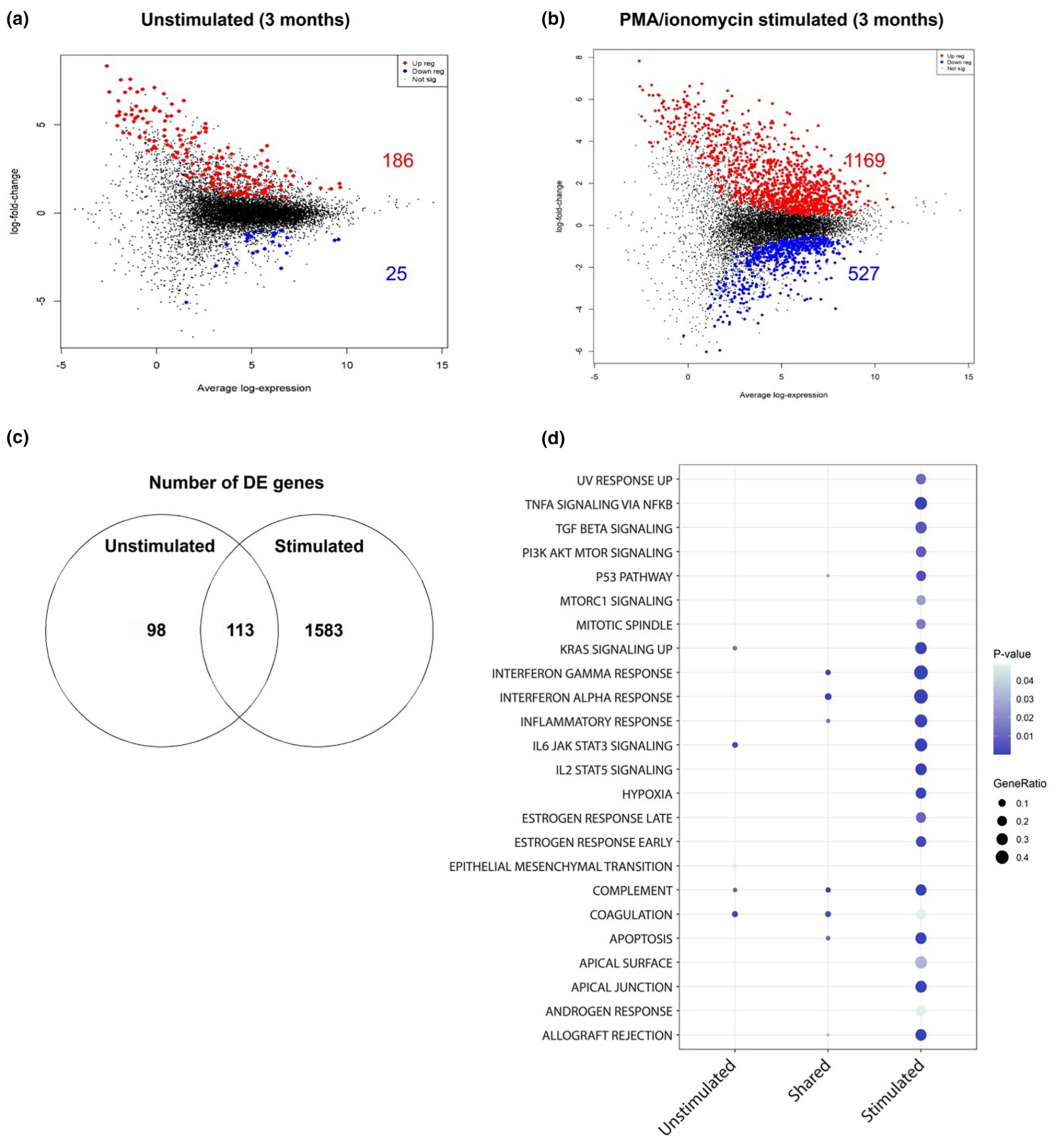
Mean difference (MD) plots in unstimulated **(a)** and PMA/ionomycin stimulated **(b)** patient samples, at 3 months, who developed infection compared to those who did not develop infection (full list Supplementary table 4). Genes were considered differentially expressed based on an adjusted *P*‐value < 0.05. Differentially expressed genes (DEGs) that correlated with subsequent infection were grouped into present in unstimulated samples‐only, across both (shared), or in stimulated‐only **(c)** and subsequently grouped into hallmark gene sets **(d)**. Infection‐free *n* = 8, infection *n* = 3.

However, in accordance with the protein data, patients at 3 months who developed subsequent infection exhibited DEGs in both unstimulated and stimulated PBMC cultures. In unstimulated samples, 186 genes were upregulated and 25 downregulated in patients who subsequently developed infection (Figure 4a). In the PMA/ionomycin stimulated samples, 1169 genes were upregulated and 527 genes were downregulated in patients who developed subsequent infection (Figure 4b), compared to stimulated patients who did not develop infection. We next performed pathway analysis of DEGs in unstimulated samples at 3 months (Figure 4c). We observed a distinct inflammatory transcriptional profile, including upregulation of coagulation, complement, IL‐6/JAK/STAT3 signalling, apoptosis, interferon and inflammatory response, as well as IL‐2/STAT5 signalling. Analysis of stimulated samples (Figure 4d) showed a similar, albeit amplified, inflammatory response involving related pathways, such as IL‐6/JAK/STAT3 signalling, interferon and inflammatory responses, IL‐2/STAT5 signalling, complement, apoptosis, TNFa signalling and reactive oxygen species. We then compared the differences in gene expression associated with infection in both unstimulated and stimulated patient samples (Figure 4e). There were 98 unique DEGs in unstimulated samples, with 1583 DEGs unique to the PMA/ionomycin stimulated group. There were 113 DEGs common amongst both groups. From these 113 conserved genes, the hallmarks interferon alpha, interferon gamma, complement, coagulation, apoptosis, inflammatory response, p53 pathway and allograft rejection were all upregulated, with no hallmarks downregulated.

**Figure 5:**
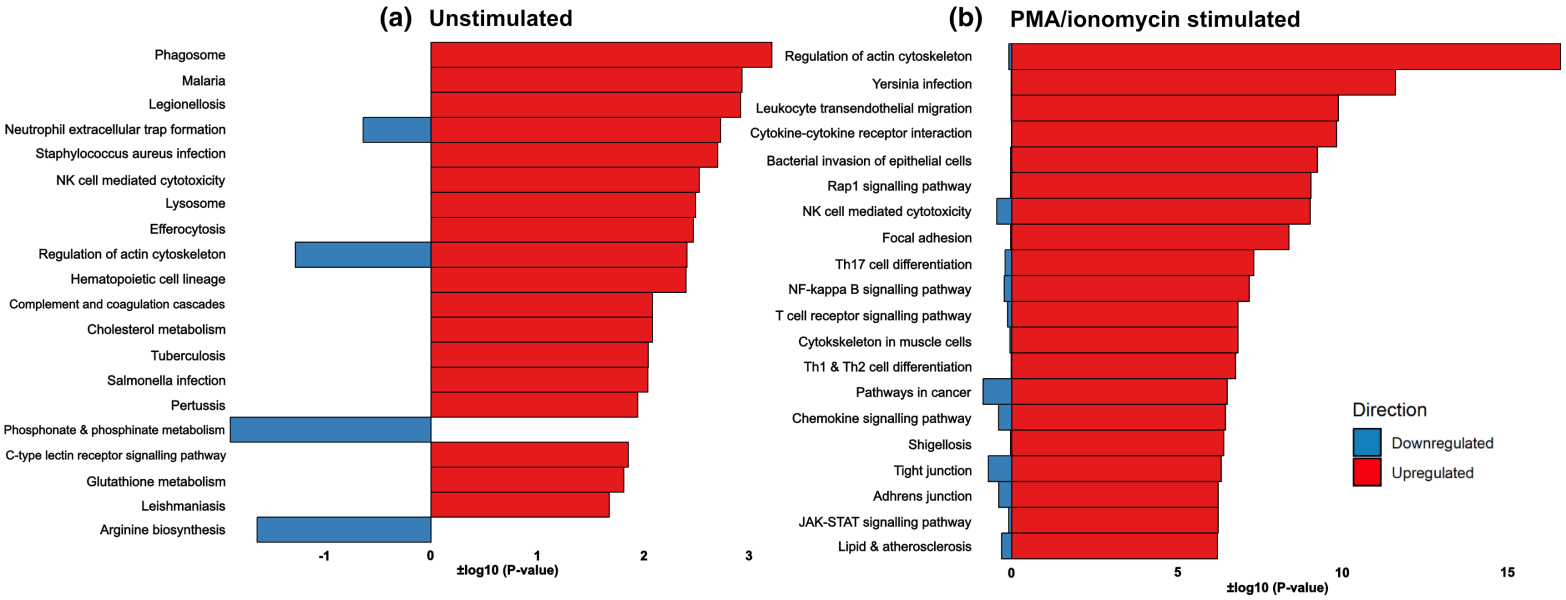
Kyoto Encyclopedia of Genes and Genomes (KEGG) pathway enrichment in unstimulated **(a)** and PMA/ionomycin stimulated **(b)** patient samples, at 3 months, who developed infection compared to those who did not develop infection. Genes were considered differentially expressed based on an adjusted *P*‐value. *P* < 0.05. The pathway analysis was limited to the top 20 most significantly enriched pathways. Infection‐free *n* = 8, infection *n* = 3.

To determine the extent of overlap between protein and RNA expression, we utilised Kyoto Encyclopedia of Genes and Genomes (KEGG) pathway enrichment of the bulk RNAseq datasets on patients at 3 months, comparing patients that remained infection‐free to those who developed infection (Figure 5). In unstimulated samples, multiple pathways associated with infection were upregulated. These include the malaria, Legionellosis, *Staphylococcus aureus* infection, tuberculosis, Salmonella infection, Pertussis and Leishmaniasis KEGG pathways, highlighting that genes and pathways with critical roles for immune responses against a broader spectrum of pathogens are dysregulated in this patient group. In stimulated samples, there was upregulation of pathways associated with immune cell signalling in patients that developed an infection within 3 months of sampling compared to patients that remained infection‐free. These included leukocyte trans‐endothelial migration, cytokine–cytokine receptor interaction, Th17 cell differentiation, NF‐kappa B signalling pathway, T‐cell receptor signalling pathway, and Th1 and Th2 cell differentiation. Interestingly, the enrichment of genes associated with natural killer cell‐mediated cytotoxicity was upregulated in patients that later developed an infectious period, regardless of stimulation.

Additionally, we also performed KEGG enrichment analysis of the seven proteins (IL‐10, IL‐31, ENA‐78, fractalkine, MCP‐2, HGF and MMP‐1) at 3 months that were associated with subsequent infection, which assigned them to several pathways that were also dysregulated in the above transcriptome analysis. Specifically, proteins associated with the Pertussis, cytokine‐cytokine receptor interaction and chemokine signalling pathways were enriched. However, protein enrichment also demonstrated upregulation of the IL‐17 signalling pathway, TNF signalling pathway, and viral protein interaction with cytokine and cytokine receptor. Whilst not identically overlapping, these are very closely linked with the Th17 cell differentiation, NF‐Kappa B signalling pathway, and Th1 and Th2 cell differentiation pathways upregulated in stimulated RNA transcriptomics.

## Discussion

Infection remains a leading cause of morbidity and mortality in patients with CLL.^2^, ^4^ Rates of infection are highest in the first 12 months from commencing targeted BTK inhibitor therapy and are even more prevalent in relapsed or refractory patients.^5^, ^6^ Despite being an effective treatment, targeted therapies have complicated clinical risk assessment for infections for these patients because of the less predictable interaction between patient, disease and treatment‐related risk factors. As such, it has been challenging to identify high‐risk CLL patients who require clinical prevention measures. Previous studies have demonstrated comprehensive immune profiling can identify MM patients who are at risk of infection.^8^, ^9^ We aimed to utilise the same approach in relapsed refractory patients with CLL and treated with ibrutinib to identify immune profiles associated with future infection risk as proof of concept for this cohort in the setting of targeted therapies.

Our immune profiling approach consisted of determining cell populations, protein and gene expression. We established that ibrutinib had no effect on relative cell populations across the 6 months included in this study. Similarly, there were no associations of cell counts with subsequent risk of infection. Overall, our pilot data suggest that relative cell population/percentages are not a useful predictor of infection risk for future CLL studies. This is in line with similar studies in patients with MM.^8^, ^9^

Protein signatures showed immune responses to PMA/ionomycin were suppressed over time. This suggests that ibrutinib therapy significantly disrupts the ability of PBMCs to respond to pro‐inflammatory agents. Interestingly, whilst whole protein profiles did not correlate to risk of infection before commencing therapy, our data demonstrate whole protein profiles in response to PMA/ionomycin stimulation can be used to differentiate risk of infection after 3 months of ibrutinib therapy. This effect was also observed in patients at 6 months, but at a lesser magnitude. At both timepoints, patients who subsequently developed infection demonstrated higher whole protein responses to PMA/ionomycin stimulation than patients who did not develop infection. Upon further investigation, we found that specific cytokines, chemokines and growth factors could be correlated with subsequent infection: IL‐10, IL‐31, ENA‐78, fractalkine, MCP‐2, HGF and MMP‐1. While all these candidate molecules warrant further investigation across further cohorts, together these data suggest that the measurement of the blood immune protein response to PMA/ionomycin stimulation may hold promise as a predictive tool for subsequent risk for infection in CLL patients on targeted BTK inhibitor immune therapies, such as ibrutinib.

Transcriptome profiles demonstrated that PMA and ionomycin stimulation upregulated multiple inflammatory hallmark gene pathways as expected. The magnitude of this response diminished after 3 months of therapy before rebounding at 6 months. Interestingly, the protein expression aligned with this paradigm at baseline and 3 months but, in contrast, remained suppressed at 6 months. This perhaps highlights a partial host equilibrium occurring with ibrutinib therapy but with substantial differences remaining between transcription and translation of immune signatures. We identified significant transcriptomic correlations with infection at 3 months following ibrutinib treatment, with patients who developed subsequent infection demonstrating upregulation of inflammatory pathways, such as IL‐6/ JAK/ STAT3 signalling, interferon gamma and alpha signalling, IL‐2/ STAT5 signalling, complement, apoptosis, TNFa signalling and reactive oxygen species. Interestingly, these inflammatory hallmarks, such as interferon alpha and gamma responses, were upregulated at 3 months prior to subsequent infection with or without stimulation. However, our pilot study was unable to discern any immune signatures associated with infection at baseline or at 6 months. This suggests that the use of transcriptomics could be more sensitive as it can detect differences in patients who develop subsequent infection without stimulating the cells in culture. In this study, we demonstrate an overlap in enrichment profiles between protein and RNA. However, there is always a level of dissonance between transcription and translation during ibrutinib therapy. We would like to clarify that while transcriptional profiling can provide useful insights, it often does not correlate well with cytokine protein abundance. This is particularly true in the context of inflammation, where cytokine expression is tightly regulated at multiple levels, including mRNA stability, translation efficiency and post‐translational processing. Given that cytokine‐mediated pathology operates at the protein level, and that secretion dynamics cannot be inferred from mRNA alone, we opted to measure both to ensure the most biologically relevant readouts. Therefore, it would be prudent to assess both proteomic and transcriptomic expressions for future immune profiling studies both big and small.

Limitations include the small cohort of relapsed refractory CLL patients and the relatively short duration of this study. This may limit the generalisation of our findings across all stages of CLL and treatment strategies. However, we have presented a representative clinical cohort of patients on standardised targeted therapy (ibrutinib) at higher risk for infection. Despite the small size, we have demonstrated a promising clinical prognostic tool that warrants further investigation amongst a larger and wider clinical cohort. Secondly, the present study utilised bulk RNA sequencing to estimate cell proportions. It must be acknowledged that this technique is not as accurate as true cell counting methods. However, our results were consistent with the literature which suggests cell counts are of little help in haematological malignancies.^14^

Compared to other approaches, such as machine learning, this pilot study has demonstrated the potential of a simplistic protein response to stimulation as a means of predicting future risk for infection. This has the potential to standalone using existing clinical tools, such as ELISAs or serotyping that are currently used for immunophenotyping, or incorporated within a larger machine learning algorithm to increase their robustness.^15^ However, there is a need to validate the utility of this approach in larger prospective cohort studies.

## Methods

### Patient population and definitions

This study is focused on a homogenous relapsed and refractory CLL patient population who have received at least one line of therapy. Patients with CLL who commenced treatment for relapse or refractory disease with the BTK inhibitor ibrutinib at Peter MacCallum Cancer Centre (PMCC) from 2014 to 2018 were evaluated. Blood samples from CLL patients participating in a prospective study of changes in tumor and host immunity were collected at pre‐defined intervals and utilised for this study. As per institutional guidelines, antimicrobial (bacterial, viral and fungal) prophylaxis was not routinely used in the setting of targeted therapies.

Clinical and microbiology records were reviewed to capture patient demographics, CLL characteristics and characteristics of infection episodes. Episodes of infection were defined and classified as microbiologically defined (MDI) or clinically defined infections (CDI) according to international consensus reporting standards.^16^ In brief, MDI consisted of infection episodes with a clinical syndrome and compatible pathogen(s) isolated on microbiological testing whilst CDI consists of clinical syndrome of infectious origin but no compatible microbiology on testing. Severity of infection was graded according to the common terminology criteria for adverse events. Subsequent infection was defined by patients who developed an infection episode within 3 months following a sample collection, that is ‘Baseline’—infective episode between 0 and 3 months, ‘3 months’—infective episode between 3 and 6 months, and ‘6 months’—infective episode between 6 and 9 months.

This study (17/182) and use of collected samples (Project 13/36) were approved by the Peter MacCallum Cancer Centre Human Research Ethics Committee (HREC/17/PMCC/209).

### Sample collection and processing

Blood samples were collected from patients before commencing BTK therapy as well as from patients after 3 and 6 months of ibrutinib treatment. It was then documented whether the patient experienced an infectious episode in the 3 months after that time point. In total, this encompasses the first 9 months of ibrutinib therapy. This was chosen to allow the highest resolution during the period of time when patients are both most likely to acquire an infection^12^, ^17^, ^18^ and undergo changes induced by ibrutinib. Blood samples collected from CLL patients pre‐commencement of BTK inhibitor therapy were utilised as the baseline control group. Peripheral blood mononuclear cells were isolated by Ficoll paque (Sigma‐Aldrich, St. Louis, USA) density separation and cryopreserved in liquid nitrogen until required for further analysis.

PBMCs were thawed in pre‐warmed thawing medium (RPMI + 10% FBS + Benzonase; Sigma‐Aldrich), washed twice and rested in RPMI supplemented with 10% (vol/vol) FBS for 2 h at 37°C. Cells were either re‐stimulated with PMA and ionomycin (Sigma‐Aldrich) or left untreated for 4 h for Luminex or RNA sequencing (RNAseq).

### Protein profiling

Protein expression was quantified by ELISA using the ProcartaPlex™ Human Immune Monitoring Panel, 65‐plex (complete protein list Supplementary table 1; Invitrogen, Waltham, USA; Cat #EPX650‐10065‐901). Briefly, up to 50 μL of sample was used, incubated with magnetic capture beads, washed and incubated with detection antibodies and streptavidin according to the manufacturer's instructions. Proteins were recorded on a Luminex 200 Analyzer (Luminex, Austin, USA) and quantitated via comparison with a standard curve.

### Transcriptional profiling

PBMC RNA extraction was performed using the Isolate II RNA mini kit (Meridian Biosciences, Cincinnati, USA; Cat. #BIO‐52072). An input of 100 ng of total RNA was prepared and indexed separately for Illumina sequencing using the TruSeq RNA Sample Prep Kit (Illumina, San Diego, USA; Cat. #RS‐122‐2001) as per the manufacturer's instructions. Each library was quantified using RNA and DNA ScreenTapes (Agilent, Santa Clara, USA; Cat. #5067–5576 and #5067–5582) on the 2200 TapeStation system (Agilent). The indexed libraries were pooled for single‐end sequencing (1 × 75 cycles) on a NextSeq 500 instrument using the v2 150cycle High Output kit (Illumina; Cat. #20024906) as per the manufacturer's instructions with a coverage of 30 M reads per sample. The base calling and quality scoring were determined using the Real‐Time Analysis onboard software v2.4.6, whilst the FASTQ file generation and demultiplexing utilised the bcl2fastqconversion software v2.15.0.4 and processed through FastQC and MultiQC for quality control.

All reads were aligned to the build hg38 human genome using align from the Rsubread software package v2.4.3.^19^ Between 86.7% and 99.5% of reads were successfully mapped to the human reference genome. The number of reads overlapping genes was summarised into counts using featureCounts from Rsubread.^20^ An average of 68.4% of reads were assigned to genes across all samples. Genes were identified using GENCODE annotation for the human genome v37.^21^ Differential expression (DE) analyses were then undertaken using the edgeR^22^ and limma^23^ software packages v3.40.2 and v3.54.2, respectively.

Prior to analysis, haemoglobin genes, ribosomal RNAs, non‐protein coding immunoglobulin genes and those labelled in the annotation as ‘to be experimentally confirmed’ were removed. Gender‐specific genes, including XIST and those unique to the Y‐chromosome were also removed to avoid gender bias. Expression‐based filtering for lowly expressed genes was performed such that only genes with a minimum counts per million mapped reads (CPM) of 17 in at least three samples were retained for downstream analysis. Following filtering, 11 265 genes remained. Library sizes were then normalised using the trimmed mean of M‐values method.

Following filtering and normalisation, the data were transformed to log_2_‐CPM with associated precision weights using voom and the correlation between samples from the same patient estimated using limma's duplicateCorrelation function. Sample‐specific weights were calculated using limma's voomQualityWeights.^24^ Differential expression was then assessed using linear models and robust empirical Bayes moderated *t*‐statistics. To increase precision, the linear models included the patient correlation estimate and sample weights. The false discovery rate (FDR) was controlled below 5% using the Benjamini and Hochberg method. Analyses of the Gene Ontology (GO) terms and KEGG pathways were performed using limma's goana and kegga functions, respectively. The analysis of the Hallmark gene sets from the Molecular Signatures Database was achieved using limma's fry function. Deconvolution of bulk RNAseq data to identify immune cell subsets was performed using dtangle.^13^

The mean difference (MD) plots were generated using limma's plotMD function and the heatmaps using limma's coolmap function.

### Statistical analysis

Categorical variables were summarised as proportions whilst continuous variables were summarised with median and interquartile range (IQR). Quantitative analysis for cell populations and protein expression was conducted using one‐ or two‐way ANOVA if data adhered to normality, while the Kruskal–Wallis test was used for non‐parametric datasets. Adjusted *P*‐values were determined using the Bonferroni *post hoc* method where applicable. Statistical analysis was completed using Prism v9 (Prism) with *P* < 0.05 considered statistically significant.

## Author contributions

**Lewis J Williams:** Data curation; formal analysis; investigation; methodology; project administration; validation; visualization; writing – original draft; writing – review and editing. **Connie SN Li‐Wai‐Suen:** Data curation; formal analysis. **Alex L Garnham:** Data curation; formal analysis. **Stefanie M Bader:** Writing – review and editing. **Constantine S Tam:** Conceptualization; investigation; methodology. **Ashley Whitechurch:** Investigation; resources. **Monica A Slavin:** Conceptualization; funding acquisition; supervision. **Marcel Doerflinger:** Investigation; methodology; supervision; visualization; writing – review and editing; data curation; conceptualization. **Benjamin W Teh:** Conceptualization; funding acquisition; methodology; supervision; visualization; writing – review and editing.

## Conflict of interest

MAS has received grants from Gilead Sciences, Merck and F2G; sits on advisory boards for Roche, Pfizer, Cidara and Basilea; and received speaker fees from Pfizer, Gilead Sciences and F2G. AW has received honoraria from Janssen. CST has received honorarium from Janssen, BeiGene, AstraZeneca and AbbVie.

## Supporting information


Supporting information

## Data Availability

The data that support the findings of this study are available on request from the corresponding author. The data are not publicly available due to privacy or ethical restrictions.
