# Supplementary material for: Elucidating novel immune profiles for predicting infection in high‐risk cohorts: a pilot study in patients with relapsed and refractory chronic lymphocytic leukaemia
Source: Clin Transl Immunology. 2025 Aug 3;14(8):e70049. doi: 10.1002/cti2.70049 (PMC12318685; doi:10.1002/cti2.70049)
Supplement: Supplementary file 1 — Supporting information [file CTI2-14-e70049-s001.docx]

# SUPPORTING INFORMATION

**Supplementary table 1.** Complete list of proteins included in the 65-plex immune response Luminex.

| ***ProcartaPlex™ Human Immune Monitoring Panel 65plex Cat#* *EPX650-10065-901*** | | |
| --- | --- | --- |
| G-CSF | IL-16 | M-CSF |
| BLC (CXCL13) | IL-17A (CTLA-8) | MCP-1 (CCL2) |
| APRIL | IL-18 | MCP-2 (CCL8) |
| BAFF | IL-1alpha | MCP-3 (CCL7) |
| CD30 | IL-1beta | MDC (CCL22) |
| CD40L (CD154) | IL-2 | MIF |
| ENA-78 (CXCL5) | IL-20 | MIG (CXCL9) |
| Eotaxin (CCL11) | IL-21 | MIP-1 alpha(CCL3) |
| Eotaxin-2 (CCL24) | IL-22 | MIP-1 beta (CCL4) |
| Eotaxin-3 (CCL26) | IL-23 | MIP-3 alpha (CCL20) |
| FGF-2 | IL-27 | MMP-1 |
| Fractalkine (CX3CL1) | IL-2R (CD25) | NGF beta |
| GM-CSF | IL-3 | SCF |
| Gro-alpha (CXCL1) | IL-31 | SDF-1alpha (CXCL12) |
| HGF | IL-4 | TNF-RII |
| I-TAC (CXCL11) | IL-5 | TNF alpha |
| IFN alpha | IL-6 | TNF beta |
| IFN gamma | IL-7 | TRAIL (CD253) |
| IL-10 | IL-8 (CXCL8) | TSLP |
| IL-12p70 | IL-9 | TWEAK |
| IL-13 | IP-10 (CXCL10) | VEGF-A |
| IL-15 | LIF |  |

**Supplementary table 2.** Complete list of significantly different hallmark gene sets for unstimulated samples at 3 months compared to unstimulated baseline.

| **Hallmark gene set** | **No. Genes** | **Direction** | **P-value** |
| --- | --- | --- | --- |
| SPERMATOGENESIS | 44 | Down | 0.01245 |
| PROTEIN SECRETION | 87 | Down | 0.01277 |
| G2M CHECKPOINT | 149 | Down | 0.02882 |
| PI3K AKT MTOR SIGNALING | 86 | Down | 0.02897 |
| MYOGENESIS | 94 | Up | 0.04831 |
| BILE ACID METABOLISM | 59 | Up | 0.04967 |

**Supplementary table 3.** Complete table of hallmark pathways significantly up- or downregulated in response to PMA/ ionomycin at Baseline and after 3 or 6 months of Ibrutinib treatment.

| **Hallmark gene set** | **No. Genes** | **Direction** | **P-value** |
| --- | --- | --- | --- |
| Baseline | | | |
| ADIPOGENESIS | 150 | Down | 0.003930 |
| ANDROGEN RESPONSE | 79 | Up | 0.009207 |
| APICAL SURFACE | 18 | Down | 0.003300 |
| BILE ACID METABOLISM | 59 | Down | 0.000346 |
| FATTY ACID METABOLISM | 115 | Down | 0.000068 |
| HEME METABOLISM | 140 | Down | 0.001344 |
| ­_­­­­­­­­_ IL2 STAT5 SIGNALING | 160 | Up | 0.013363 |
| INFLAMMATORY RESPONSE | 137 | Up | 0.011867 |
| MTORC1 SIGNALING | 178 | Up | 0.002974 |
| MYC TARGETS V1 | 191 | Up | 0.000377 |
| MYC TARGETS V2 | 55 | Up | 0.000034 |
| NOTCH SIGNALING | 23 | Up | 0.008117 |
| PEROXISOME | 77 | Down | 0.000494 |
| TGF BETA SIGNALING | 44 | Up | 0.004897 |
| TNFA SIGNALING VIA NFKB | 176 | Up | 0.000039 |
| UNFOLDED PROTEIN RESPONSE | 108 | Up | 0.000499 |
| UV RESPONSE UP | 115 | Up | 0.007284 |
| 3 months | | | |
| ANDROGEN RESPONSE | 79 | Up | 0.00841677 |
| APOPTOSIS | 130 | Up | 0.00366538 |
| BILE ACID METABOLISM | 59 | Down | 0.00041264 |
| HEME METABOLISM | 140 | Down | 0.00609911 |
| IL6 JAK STAT3 SIGNALING | 67 | Up | 0.00728893 |
| INFLAMMATORY RESPONSE | 137 | Up | 0.00948112 |
| MTORC1 SIGNALING | 178 | Up | 0.0004396 |
| MYC TARGETS V1 | 191 | Up | 5.34E-05 |
| MYC TARGETS V2 | 55 | Up | 0.00054419 |
| PEROXISOME | 77 | Down | 0.01279647 |
| REACTIVE OXYGEN SPECIES PATHWAY | 45 | Up | 0.00075062 |
| TGF BETA SIGNALING | 44 | Up | 0.00124801 |
| TNFA SIGNALING VIA NFKB | 176 | Up | 7.45E-06 |
| UNFOLDED PROTEIN RESPONSE | 108 | Up | 0.00194909 |
| UV RESPONSE UP | 115 | Up | 2.65E-05 |
| WNT BETA CATENIN SIGNALING | 28 | Up | 0.01462352 |
| 6 months | | | |
| ALLOGRAFT REJECTION | 168 | Up | 0.00086597 |
| ANDROGEN RESPONSE | 79 | Up | 4.79E-06 |
| ANGIOGENESIS | 16 | Up | 0.04297344 |
| APOPTOSIS | 130 | Up | 6.90E-06 |
| BILE ACID METABOLISM | 59 | Down | 0.00016901 |
| COMPLEMENT | 143 | Up | 0.03065574 |
| EPITHELIAL MESENCHYMAL TRANSITION | 91 | Up | 0.00431764 |
| ESTROGEN RESPONSE EARLY | 109 | Up | 0.04933717 |
| ESTROGEN RESPONSE LATE | 105 | Up | 0.01881735 |
| FATTY ACID METABOLISM | 115 | Down | 0.01422235 |
| HEME METABOLISM | 140 | Down | 0.00210603 |
| IL2 STAT5 SIGNALING | 160 | Up | 3.84E-05 |
| IL6 JAK STAT3 SIGNALING | 67 | Up | 8.72E-06 |
| INFLAMMATORY RESPONSE | 137 | Up | 4.80E-06 |
| INTERFERON GAMMA RESPONSE | 178 | Up | 0.00364954 |
| MTORC1 SIGNALING | 178 | Up | 9.36E-05 |
| MYC TARGETS V1 | 191 | Up | 7.60E-05 |
| MYC TARGETS V2 | 55 | Up | 9.97E-05 |
| NOTCH SIGNALING | 23 | Up | 0.00064203 |
| P53 PATHWAY | 164 | Up | 0.02729903 |
| PEROXISOME | 77 | Down | 0.003698 |
| REACTIVE OXYGEN SPECIES PATHWAY | 45 | Up | 0.000577 |
| TGF BETA SIGNALING | 44 | Up | 1.10E-05 |
| TNFA SIGNALING VIA NFKB | 176 | Up | 6.32E-09 |
| UNFOLDED PROTEIN RESPONSE | 108 | Up | 0.00055882 |
| UV RESPONSE UP | 115 | Up | 0.00010536 |
| WNT BETA CATENIN SIGNALING | 28 | Up | 0.0048358 |

**Supplementary table 4.** A complete list of significantly expressed hallmark gene sets in individuals at 3 months who acquired infection compared to those who did not.

| Unstimulated | | | | PMA/ionomycin stimulation | | | |
| --- | --- | --- | --- | --- | --- | --- | --- |
| Hallmark | NGenes | Direction | Adj. *P*-value | Hallmark | NGenes | Direction | Adj. *P*-value |
| COAGULATION | 62 | Up | 4.7076E-04 | IL6 JAK STAT3 SIGNALING | 67 | Up | 4.8200E-06 |
| COMPLEMENT | 143 | Up | 4.7076E-04 | INTERFERON GAMMA RESPONSE | 178 | Up | 5.4170E-06 |
| IL6 JAK STAT3 SIGNALING | 67 | Up | 1.1931E-02 | INTERFERON ALPHA RESPONSE | 90 | Up | 3.4141E-05 |
| KRAS SIGNALING UP | 108 | Up | 1.1931E-02 | INFLAMMATORY RESPONSE | 137 | Up | 3.4141E-05 |
| EPITHELIAL MESENCHYMAL TRANSITION | 91 | Up | 1.1931E-02 | IL2 STAT5 SIGNALING | 160 | Up | 3.9851E-05 |
| ALLOGRAFT REJECTION | 168 | Up | 1.9951E-02 | ALLOGRAFT REJECTION | 168 | Up | 4.4160E-05 |
| APOPTOSIS | 130 | Up | 1.9951E-02 | COMPLEMENT | 143 | Up | 9.3784E-05 |
| INTERFERON GAMMA RESPONSE | 178 | Up | 1.9951E-02 | APOPTOSIS | 130 | Up | 1.1191E-04 |
| INFLAMMATORY RESPONSE | 137 | Up | 1.9951E-02 | WNT BETA CATENIN SIGNALING | 28 | Up | 1.2235E-04 |
| INTERFERON ALPHA RESPONSE | 90 | Up | 1.9951E-02 | KRAS SIGNALING UP | 108 | Up | 3.2272E-04 |
| UV RESPONSE DN | 89 | Up | 3.3253E-02 | COAGULATION | 62 | Up | 5.6475E-04 |
| APICAL JUNCTION | 108 | Up | 3.3253E-02 | APICAL JUNCTION | 108 | Up | 2.4337E-03 |
| HEDGEHOG SIGNALING | 21 | Up | 4.8039E-02 | EPITHELIAL MESENCHYMAL TRANSITION | 91 | Up | 2.8772E-03 |
| IL2 STAT5 SIGNALING | 160 | Up | 4.8039E-02 | TNFA SIGNALING VIA NFKB | 176 | Up | 2.9121E-03 |
| MYOGENESIS | 94 | Up | 4.8039E-02 | TGF BETA SIGNALING | 44 | Up | 3.2642E-03 |
| HYPOXIA | 130 | Up | 4.8759E-02 | ANGIOGENESIS | 16 | Up | 3.9684E-03 |
| APICAL SURFACE | 18 | Up | 7.5503E-02 | REACTIVE OXYGEN SPECIES PATHWAY | 45 | Up | 8.1559E-03 |
| REACTIVE OXYGEN SPECIES PATHWAY | 45 | Up | 8.7195E-02 | HEDGEHOG SIGNALING | 21 | Up | 2.2089E-02 |
| BILE ACID METABOLISM | 59 | Up | 8.9619E-02 | NOTCH SIGNALING | 23 | Up | 3.0275E-02 |
| XENOBIOTIC METABOLISM | 109 | Up | 9.4377E-02 | PI3K AKT MTOR SIGNALING | 86 | Up | 3.4860E-02 |
| PEROXISOME | 77 | Up | 9.9512E-02 | MYOGENESIS | 94 | Up | 4.5702E-02 |
| ANGIOGENESIS | 16 | Up | 1.2294E-01 | UV RESPONSE DN | 89 | Up | 4.8662E-02 |
| UNFOLDED PROTEIN RESPONSE | 108 | Down | 1.6761E-01 | P53 PATHWAY | 164 | Up | 4.8662E-02 |
| PANCREAS BETA CELLS | 12 | Up | 1.7714E-01 | ANDROGEN RESPONSE | 79 | Up | 7.4083E-02 |
| P53 PATHWAY | 164 | Up | 1.7714E-01 | HYPOXIA | 130 | Up | 7.5005E-02 |
| PI3K AKT MTOR SIGNALING | 86 | Up | 1.7714E-01 | PANCREAS BETA CELLS | 12 | Up | 8.1392E-02 |
| ESTROGEN RESPONSE LATE | 105 | Up | 1.7892E-01 | UV RESPONSE UP | 115 | Up | 1.4493E-01 |
| CHOLESTEROL HOMEOSTASIS | 60 | Up | 1.7892E-01 | ESTROGEN RESPONSE LATE | 105 | Up | 1.6912E-01 |
| WNT BETA CATENIN SIGNALING | 28 | Up | 2.1638E-01 | KRAS SIGNALING DN | 53 | Up | 2.0223E-01 |
| TGF BETA SIGNALING | 44 | Up | 2.3111E-01 | XENOBIOTIC METABOLISM | 109 | Up | 2.1489E-01 |
| E2F TARGETS | 151 | Down | 2.8455E-01 | SPERMATOGENESIS | 44 | Down | 3.2810E-01 |
| GLYCOLYSIS | 127 | Up | 2.8455E-01 | ESTROGEN RESPONSE EARLY | 109 | Up | 3.7226E-01 |
| MYC TARGETS V2 | 55 | Down | 3.1388E-01 | PROTEIN SECRETION | 87 | Down | 4.2702E-01 |
| KRAS SIGNALING DN | 53 | Up | 3.4552E-01 | GLYCOLYSIS | 127 | Up | 4.2702E-01 |
| NOTCH SIGNALING | 23 | Up | 3.8581E-01 | MTORC1 SIGNALING | 178 | Up | 5.3160E-01 |
| ANDROGEN RESPONSE | 79 | Up | 4.0383E-01 | E2F TARGETS | 151 | Down | 5.5958E-01 |
| MYC TARGETS V1 | 191 | Down | 4.7110E-01 | APICAL SURFACE | 18 | Up | 5.6658E-01 |
| TNFA SIGNALING VIA NFKB | 176 | Up | 5.6360E-01 | MYC TARGETS V2 | 55 | Up | 6.1857E-01 |
| ESTROGEN RESPONSE EARLY | 109 | Up | 5.8178E-01 | CHOLESTEROL HOMEOSTASIS | 60 | Up | 6.2942E-01 |
| ADIPOGENESIS | 150 | Up | 6.1198E-01 | MYC TARGETS V1 | 191 | Up | 6.8203E-01 |
| G2M CHECKPOINT | 149 | Down | 6.3366E-01 | HEME METABOLISM | 140 | Down | 7.1022E-01 |
| HEME METABOLISM | 140 | Up | 6.6199E-01 | FATTY ACID METABOLISM | 115 | Down | 7.6441E-01 |
| MTORC1 SIGNALING | 178 | Down | 6.8535E-01 | OXIDATIVE PHOSPHORYLATION | 192 | Up | 7.6824E-01 |
| SPERMATOGENESIS | 44 | Down | 6.8535E-01 | PEROXISOME | 77 | Up | 8.2023E-01 |
| FATTY ACID METABOLISM | 115 | Up | 6.8595E-01 | ADIPOGENESIS | 150 | Down | 8.2023E-01 |
| DNA REPAIR | 135 | Down | 6.8883E-01 | DNA REPAIR | 135 | Down | 8.9035E-01 |
| UV RESPONSE UP | 115 | Up | 7.2635E-01 | BILE ACID METABOLISM | 59 | Down | 9.1103E-01 |
| OXIDATIVE PHOSPHORYLATION | 192 | Up | 7.5206E-01 | UNFOLDED PROTEIN RESPONSE | 108 | Down | 9.4042E-01 |
| MITOTIC SPINDLE | 161 | Up | 9.0819E-01 | MITOTIC SPINDLE | 161 | Up | 9.4042E-01 |
| PROTEIN SECRETION | 87 | Down | 9.5322E-01 | G2M CHECKPOINT | 149 | Up | 9.4042E-01 |


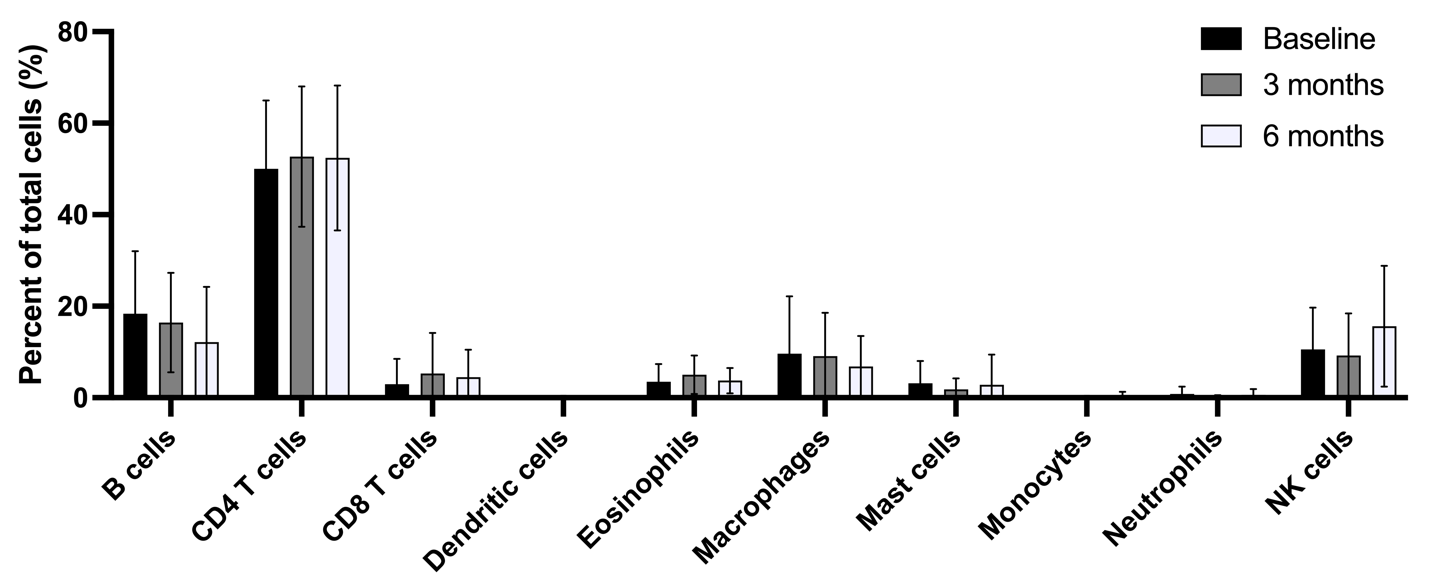


**Supplementary figure 1.** Relative cell populations derived from transcriptomic signatures in CLL patients sampled before commencing therapy (baseline) or 3 and 6 months after commencing ibrutinib treatment. Ibrutinib had no effect on relative cell populations (*P* > 0.05).

*
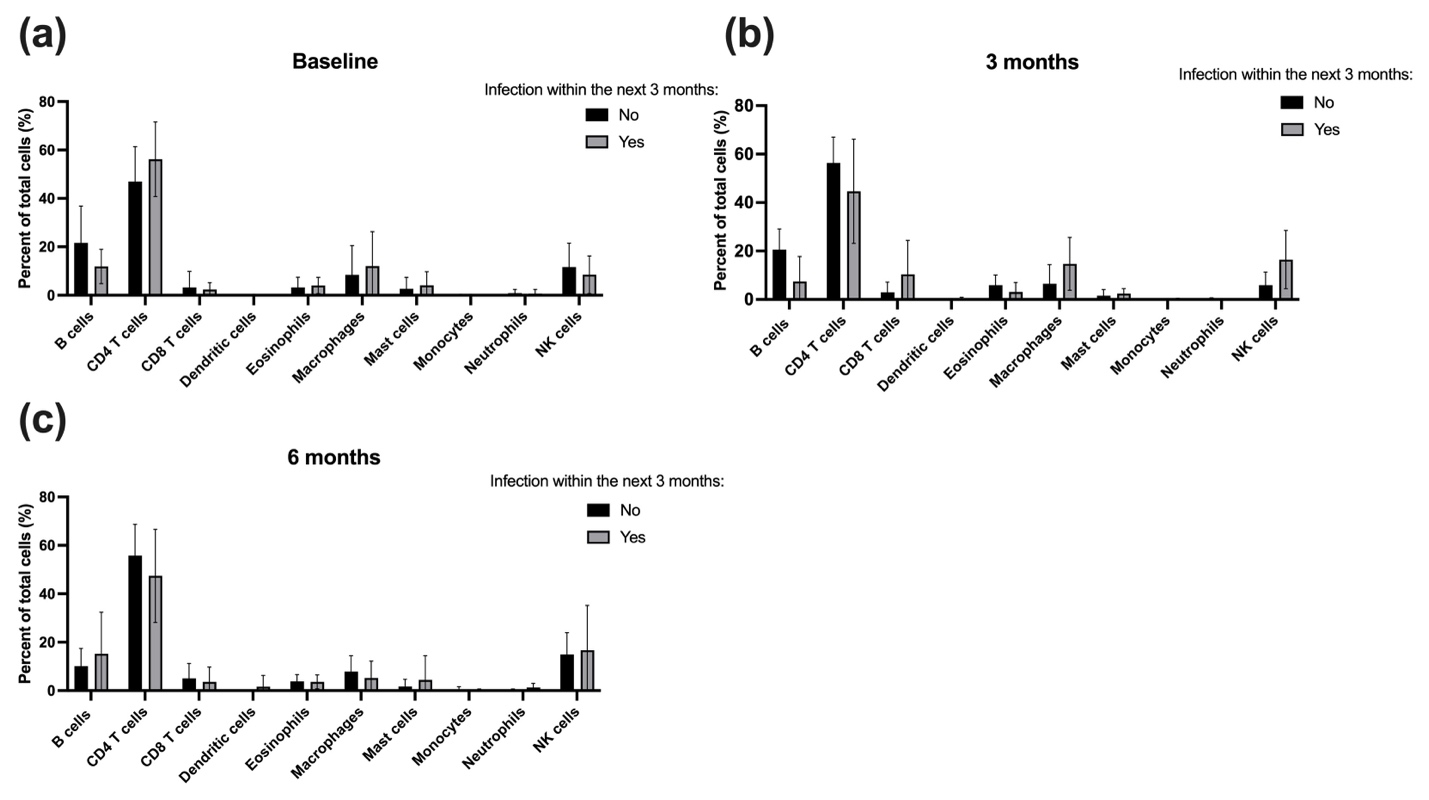
*

**Supplementary figure 2.** Relative cell populations derived from transcriptomic signatures in CLL patients sampled before (**a**; baseline) commencing therapy or 3 **(b)** and 6 months **(c)** after commencing ibrutinib treatment. Patients were divided into those who developed infection within 3 months from sample collection, and those who did not. There was no correlation between differential cell abundance and subsequent risk for infection (*P* > 0.05).
